# Associação da Ativação Endotelial e do Índice de Estresse com Risco de Doença Cardiovascular e Mortalidade por Todas as Causas em Pacientes com Osteoartrite

**DOI:** 10.36660/abc.20250012

**Published:** 2025-07-10

**Authors:** Ruoyan Li, Chaoqun Feng, Kexin Lin, Nan Wang, Xiaohong Fan

**Affiliations:** 1 Hospital of Chengdu University of Traditional Chinese Medicine Department of Orthopedics Chengdu China Hospital of Chengdu University of Traditional Chinese Medicine – Department of Orthopedics, Chengdu, Sichuan – China

**Keywords:** Osteoartrite, Doenças Cardiovasculares, Mortalidade

## Abstract

**Fundamento:**

A osteoartrite é um tipo prevalente de artrite caracterizada por alterações degenerativas crônicas no sistema músculo-esquelético, que podem resultar em danos articulares e dor crônica.

**Objetivo:**

Este estudo investigou as associações entre o índice de ativação e estresse endotelial (EASIX) e o aumento do risco de doença cardiovascular aterosclerótica (DCVA) e mortalidade por todas as causas entre pacientes diagnosticados com osteoartrite.

**Métodos:**

O estudo de coorte abrangeu 2.028 indivíduos com idades entre 40 e 79 anos com osteoartrite, utilizando dados do banco de dados do National Health and Nutrition Examination Surveys (NHANES) abrangendo os anos de 2007 a 2018. O modelo de regressão logística ponderada univariada e o modelo de Cox ponderado foram estabelecidos, respectivamente, para rastrear possíveis fatores de confusão. Um nível de significância de p < 0,05 foi adotado para todas as análises estatísticas.

**Resultados:**

O estudo revelou um risco elevado de DCVA em correlação com um log (EASIX) aumentado (razão de chances: 1,94, com intervalo de confiança de 95%: 1,57-2,41). Quando comparados a indivíduos com log (EASIX) < -1,29, aqueles com log (EASIX)>-0,78 demonstraram um risco aumentado de DCVA (razão de chances: 2,31, com intervalo de confiança de 95%: 1,68-3,18). Um valor de log (EASIX) mais alto também foi associado a um risco aumentado de mortalidade por todas as causas (razão de risco: 1,59, com intervalo de confiança de 95%: 1,14-2,23). Entre os indivíduos diagnosticados com osteoartrite, aqueles que apresentaram log (EASIX)>-0,78 apresentaram maior risco de morrer por qualquer causa, em comparação aos pacientes com log (EASIX) <-1,29.

**Conclusão:**

A presença de um alto índice EASIX foi associada a um risco aumentado de DCVA e mortalidade por todas as causas entre pacientes com osteoartrite.

## Graphical abstract

**Figura Central:**
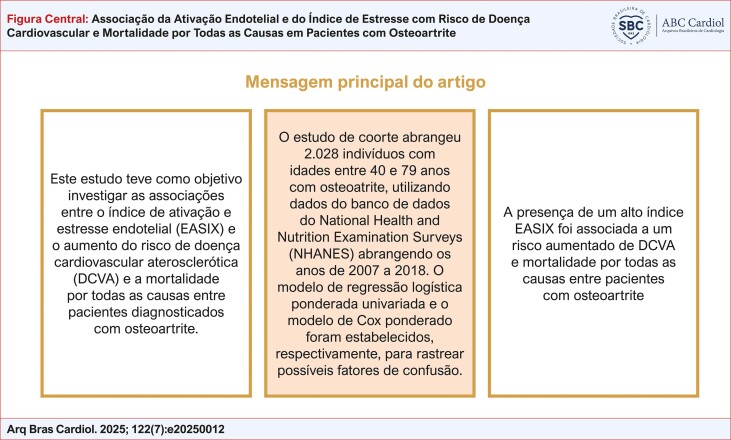
: Associação da Ativação Endotelial e do Índice de Estresse com Risco de Doença Cardiovascular e Mortalidade por Todas as Causas em Pacientes com Osteoartrite

## Introdução

A osteoartrite (OA) é uma doença articular degenerativa prevalente associada à dor crônica, declínio funcional e redução da qualidade de vida.^1^ Embora os tratamentos atuais se concentrem no controle dos sintomas, eles não interrompem a progressão da doença nem previnem danos nas articulações.^2^ Notavelmente, a OA é cada vez mais reconhecida como um fator de risco para doenças cardiovasculares (DCVs),^3 , 4^ que continua a ser uma das principais causas de mortalidade global.^5^ Identificar pacientes com OA com risco elevado de DCV é fundamental para melhorar o prognóstico e reduzir a carga da doença.

A disfunção endotelial, um mecanismo chave na aterosclerose e na DCV, pode estar subjacente ao aumento do risco de DCV em pacientes com OA.^6 , 7^ A inflamação crônica na OA leva à liberação de mediadores inflamatórios na circulação, promovendo dano endotelial e progressão da aterosclerose.^8^

O índice de ativação e estresse endotelial (EASIX) surgiu recentemente como um novo marcador de lesão endotelial,^9^ sugerindo sua utilidade potencial na avaliação do risco de DCV em pacientes com OA.

O escore de risco de doença cardiovascular aterosclerótica (DCVA) de 10 anos do *American College of Cardiology* e da *American Heart Association* é amplamente utilizado para avaliação de risco de DCV.^10 , 11^ Neste estudo, objetivamos avaliar a associação entre EASIX e alto risco de DCVA, bem como a mortalidade geral, em pacientes com OA, utilizando dados da *National Health and Nutrition Examination Surveys* (NHANES). Análises de subgrupos foram conduzidas para explorar o impacto da idade, gênero, etnia, índice de massa corporal (IMC) e uso de medicamentos anti-hiperlipidêmicos ( Figura Central ).

## Materiais e métodos

### Desenho do estudo e população

Este estudo de coorte incluiu 2.824 pacientes com OA com idades entre 40 e 79 anos no banco de dados NHANES de 2007 a 2018. O programa NHANES é um estudo abrangente e contínuo conduzido com o objetivo de espelhar o perfil demográfico abrangente da população civil não institucionalizada nos Estados Unidos, empregando um método sofisticado de amostragem em vários estágios com agrupamento probabilístico em seu projeto de pesquisa, que fornece insights abrangentes sobre diversos parâmetros de saúde, abrangendo escolhas de estilo de vida, níveis de aptidão física, hábitos alimentares e bem-estar mental.^12^ Em nosso estudo, foram incluídos participantes com diagnóstico de OA, com idades entre 40 e 79 anos, com dados completos para o cálculo do EASIX, com avaliação de DCVA em 10 anos e com dados completos de sobrevida no NHANES 2007-2018. Participantes com DCV autorrelatada foram excluídos ( Figura Central ).

### Covariáveis potenciais e definições

A educação foi categorizada como abaixo do nível de um diploma de ensino médio ou ter pelo menos um diploma de ensino médio, razão pobreza-renda (PIR), atividade física (MET × min/semana), IMC (kg/m^2^) e vitamina D (nmol/L) foram respectivamente dicotomizados como <1,3 ou ≥1,3, <750 ou ≥750, <25 ou ≥25 e <75 ou ≥75. Bebida, depressão, câncer, agentes anti-hiperlipidêmicos, esteroides corticais adrenais, analgésicos e relaxantes musculares foram dicotomizados como sim ou não. A razão neutrófilo-linfócito (RNL) e o Índice de Alimentação Saudável 2015 (HEI-2015) foram utilizados como variáveis contínuas.

A entrevista presencial realizada no MEC utilizou o Questionário de Saúde do Paciente-9 (PHQ-9)^13^ para avaliar os sintomas depressivos vivenciados nas duas semanas anteriores. Os entrevistados classificaram a frequência de nove sintomas depressivos (por exemplo, anedonia, humor deprimido, distúrbios do sono) em uma escala de 0 a 3 durante uma avaliação. Pontuações totais variaram de 0 a 27, com pontuações de 10 ou mais indicando sintomas depressivos clinicamente significativos.^14^ A atividade física foi convertida em consumo energético, e o consumo energético (MET × min) = equivalente metabólico sugerido (MET) × tempo de atividade esportiva correspondente (min). Vitamina D ≥ 75 nmol/L foi definida como adequada, < 75 nmol/L como inadequada.^15^ O HEI-2015 se concentra em nove grupos alimentares para consumo equilibrado (como frutas, vegetais, grãos integrais, laticínios e proteínas de diversas fontes), enfatizando também uma proporção saudável de gorduras insaturadas e saturadas. Quatro componentes (grãos refinados, sódio, açúcares adicionados e gorduras saturadas) devem ser consumidos com moderação. A ingestão de cada componente é padronizada usando o método da densidade com uma referência de 4184 kJ. Os componentes recebem uma pontuação que varia de 0 a 5 ou de 0 a 10. Somando as pontuações desses componentes para cada respondente com uma pontuação máxima possível de 100 pontos, uma pontuação geral pode ser calculada. O método da razão populacional é utilizado adequadamente para calcular as pontuações médias.^16 , 17^

#### Variável principal

**Figura 1:**
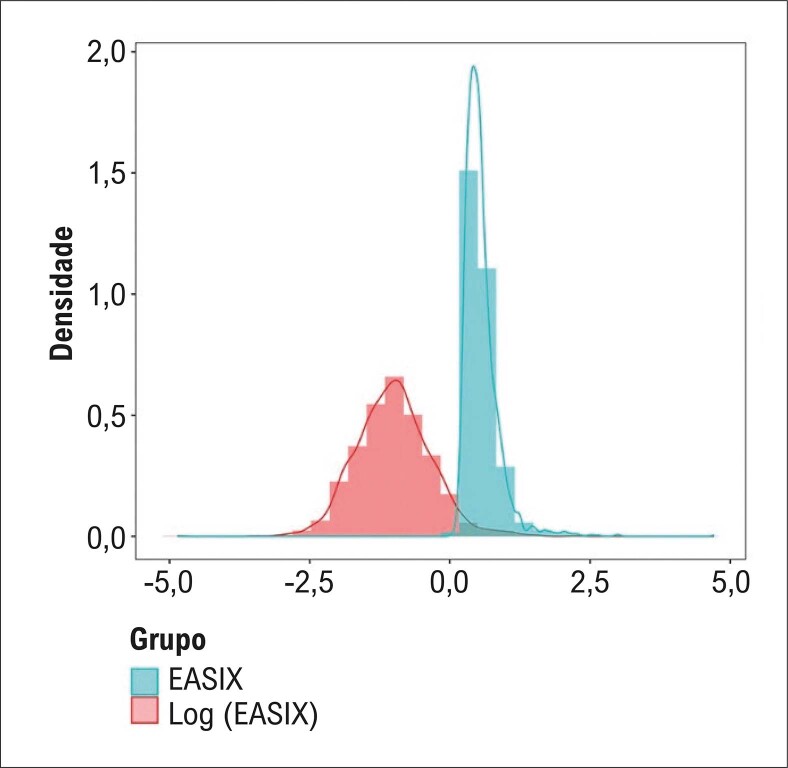
– Distribuição dos dados EASIX antes e depois da transformação log2.

A pontuação do EASIX foi a principal variável. A fórmula utilizada para calcular o EASIX foi: nível sérico de lactato desidrogenase (LDH) (U/L) × nível de creatinina (mg/dL)/contagem de plaquetas (109/L). Como o EASIX apresentou uma distribuição assimétrica, ele passou por uma transformação logarítmica antes da análise.^18^ A Figura 1 exibe os dados do EASIX antes e depois da aplicação da transformação log2.

#### Variáveis de resultado

O alto risco de DCV foi o desfecho primário, que foi avaliado pelo escore de risco de DCVA de 10 anos com base em fatores como idade, gênero e etnia. Outro exemplo seriam os níveis de colesterol, pressão arterial e uso de medicamentos.^10^ Os critérios para avaliar o risco de DCV em 10 anos classificam os indivíduos em quatro níveis de risco: baixo risco (<5%), risco limítrofe (5% a 7,5%), risco intermediário (7,5% a <20%) e alto risco (≥20%). Neste estudo, objetivamos explorar o alto risco de DCV dos pacientes.

O desfecho secundário concentrou-se na taxa de mortalidade por todas as causas entre pacientes com OA, avaliada pela vinculação de seus registros de atestado de óbito com o Índice Nacional de Óbitos (INO). A mortalidade por todas as causas refere-se ao número total de mortes ocorridas durante o período de acompanhamento, abrangendo até dezembro de 2019.

## Análise estatística

A normalidade dos dados foi avaliada pelo teste de Shapiro-Wilk, e as variáveis que não atenderam à suposição de normalidade foram transformadas adequadamente. Para comparações entre múltiplos grupos, se utilizou a Análise de Variância (ANOVA) unidirecional, seguida do teste post hoc de Tukey para identificar diferenças específicas entre os grupos quando os resultados da ANOVA foram significativos. Os dados categóricos foram apresentados como frequências e porcentagens [n (%)], e as comparações entre os grupos foram realizadas pelo teste qui-quadrado ou o método de probabilidade exata de Fisher, conforme apropriado. O estado dos valores faltantes para as variáveis é apresentado na Tabela Suplementar 1 , e imputação múltipla foi aplicada. Comparações de dados entre antes e depois de imputações múltiplas foram exibidas na Tabela Suplementar 2 . Modelo de regressão logística ponderada univariada e modelo de Cox ponderado foram estabelecidos respectivamente para rastrear possíveis fatores de confusão. Regressão logística ponderada univariada e multivariada, modelo ponderado e modelo de Cox ponderado foram aplicados respectivamente para analisar as associações do EASIX com DCVA de alto risco e mortalidade por todas as causas em pacientes com OA. A análise de subgrupo foi categorizada com base em idade, sexo, etnia, IMC e agentes anti-hiperlipidêmicos. O OR e o HR com seus respectivos IC de 95% foram calculados. A análise estatística foi realizada com SAS 9.4, com significância estatística definida em um valor de p menor que 0,05.

## Resultados

### Características dos participantes com base em diferentes pontuações do EASIX

**Figura 2:**
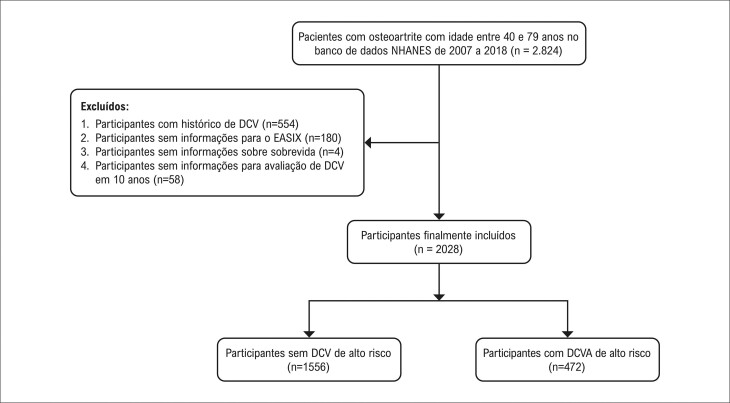
– Processo de triagem dos participantes deste estudo. DCV: doença cardiovascular; DCVA: doença cardiovascular aterosclerótica.

No total, foram identificados 2.824 pacientes com OA, com idades entre 40 e 79 anos, no banco de dados NHANES, de 2007 a 2018. Indivíduos que já haviam apresentado DCV não foram incluídos no estudo (n = 554). Participantes sem dados completos para o cálculo do EASIX (n = 180), sem dados completos de sobrevida (n = 4) ou sem a avaliação da DCVA em 10 anos (n = 58) foram excluídos. Por fim, 2.028 participantes foram incluídos, e entre eles, 472 participantes apresentavam alto risco de DCVA. O processo de triagem dos participantes é mostrado na Figura 2 .

As características dos participantes nos diferentes grupos de logaritmo (EASIX) são apresentadas na Tabela 1 . Diferenças significativas foram observadas em diversas variáveis demográficas e clínicas, incluindo idade, sexo, raça, atividade física, tabagismo, consumo de bebidas alcoólicas e comorbidades como diabetes, hipertensão e dislipidemia. Além disso, diferenças em parâmetros laboratoriais, como LDH, contagem de plaquetas e níveis de creatinina, foram observadas entre os grupos. As taxas de mortalidade e a proporção de participantes com um alto risco de DCVA também variou significativamente entre os grupos de log (EASIX).

**Tabela 1:** – Características dos participantes com diferentes níveis EASIX

Variáveis	Total (n=2028)	Log (EASIX)			p
		< -1,29 (n=676)	-1,29- -0,78 (n=663)	≥-0,78 (n=689)	
**Idade (anos), Média (DP)**	60,88 (0,31)	59,06 (0,47)	60,91 (0,49)	62,65 (0,52)***	<0,001
**Gênero, n (%)**					<0,001
Feminino	1337 (65,43)	576 (87,03)	452 (64,51)	309 (44,83)***	
Masculino	691 (34,57)	100 (12,97)	211 (35,49)	380 (55,17)***	
**Raça, n (%)**					0,008
Branco	1157 (82,90)	370 (81,67)	382 (82,42)	405 (84,61)**	
Preto	347 (6,42)	93 (5,60)	115 (6,30)	139 (7,37)**	
Outros	524 (10,68)	213 (12,73)	166 (11,28)	145 (8,02)**	
**Educação, n (%)**					0,244
No ensino médio	350 (10,07)	131 (11,69)	106 (9,07)	113 (9,49)	
Ensino médio e acima	1678 (89,93)	545 (88,31)	557 (90,93)	576 (90,51)	
**PIR, n (%)**					0,355
<1,3	483 (14,27)	166 (14.16)	170 (15,68)	147 (12,90)	
≥1,3	1545 (85,73)	510 (85,84)	493 (84,32)	542 (87,10)	
**Atividade física (MET × min/semana), n (%)**					0,015
<750	1060 (46,91)	373 (51,82)	361 (47,99)	326 (40,90)**	
≥750	968 (53,09)	303 (48,18)	302 (52,01)	363 (59,10)**	
**Tabagismo, n (%)**					0,003
Não	1697 (85,12)	535 (81,05)	552 (84,95)	610 (89,37)**	
Sim	331 (14,88)	141 (18,95)	111 (15.05)	79 (10,63)**	
**Consumo de bebida alcoólica, n (%)**					<0,001
Não	456 (17.17)	190 (22,41)	148 (15,75)	118 (13,42)***	
Sim	1176 (61,84)	391 (62,63)	388 (63,20)	397 (59,64)***	
Desconhecido	396 (20,99)	95 (14,96)	127 (21.05)	174 (26,94)***	
**Diabetes, n (%)**					0,166
Não	1528 (80,67)	498 (78,95)	514 (83,24)	516 (79,73)	
Sim	500 (19,33)	178 (21,05)	149 (16,76)	173 (20,27)	
**Hipertensão**					0,763
Não	458 (26,03)	176 (25,08)	154 (27,28)	128 (25,70)	
Sim	1570 (73,97)	500 (74,92)	509 (72,72)	561 (74,30)	
**Dislipidemia, n (%)**					0,044
Não	355 (17,67)	110 (16,27)	136 (21,40)	109 (15.20)**	
Sim	1673 (82,33)	566 (83,73)	527 (78,60)	580 (84,80)**	
**Depressão, n (%)**					0,435
Não	1800 (90,86)	586 (90,01)	589 (90,26)	625 (92,33)	
Sim	228 (9,14)	90 (9,99)	74 (9,74)	64 (7,67)	
**Câncer, n (%)**					0,018
Não	1665 (79,58)	569 (83,20)	552 (79,97)	544 (75,58)**	
Sim	363 (20,42)	107 (16,80)	111 (20.03)	145 (24,42)**	
**Agentes anti-hiperlipidêmicos**					0,002
Não	1352 (67,73)	484 (74,03)	447 (66,32)	421 (62,90)***	
Sim	676 (32,27)	192 (25,97)	216 (33,68)	268 (37,10)***	
**Esteroides corticais supra-renais, n (%)**					0,739
Não	1973 (98,06)	659 (98,40)	648 (97,74)	666 (98,05)	
Sim	55 (1,94)	17 (1,60)	15 (2,26)	23 (1,95)	
**Analgésicos, n (%)**					0,630
Não	1412 (70,43)	458 (68,85)	454 (70,70)	500 (71,73)	
Sim	616 (29,57)	218 (31.15)	209 (29,30)	189 (28,27)	
**Relaxantes musculares, n (%)**					0,528
Não	1886 (93,26)	624 (92,29)	615 (93,23)	647 (94,25)	
Sim	142 (6,74)	52 (7,71)	48 (6,77)	42 (5,75)	
**IMC (kg/m^**2**^), n (%)**					0,009
<25	389 (20.08)	144 (23,63)	130 (21.07)	115 (15,51)**	
≥25	1639 (79,92)	532 (76,37)	533 (78,93)	574 (84,49)**	
**Vitamina D (nmol/L), n (%)**					0,293
<75	909 (35,47)	303 (36,09)	315 (37,90)	291 (32,34)	
≥75	1119 (64,53)	373 (63,91)	348 (62,10)	398 (67,66)	
**RNL, Média (DP)**	2,29 (0,04)	2,24 (0,05)	2,25 (0,09)	2,38 (0,07)	0,280
**HEI 2015, Média (DP)**	53,28 (0,47)	53,01 (0,75)	53,71 (0,80)	53,10 (0,71)	0,772
**Lactato desidrogenase LDH (U/L), Média (DP)**	140,04 (0,98)	125,40 (1,40)	139,42 (1,42)	155,27 (1,64)***	<0,001
**Contagem de plaquetas (1000 células/uL), Média (DP)**	242,65 (2,30)	289,09 (3,27)	237,73 (2,54)	201,41 (2,08)***	<0,001
**Creatinina (mg/dL), Média (DP)**	0,87 (0,01)	0,72 (0,01)	0,85 (0,01)	1,05 (0,02)***	<0,001
**EASIX, Média (DP)**	0,54 (0,01)	0,32 (0,00)	0,50 (0,00)	0,82 (0,02)***	<0,001
**Log (EASIX), Média (DP)**	-1,02 (0,02)	-1,69 (0,02)	-1,02 (0,01)	-0,35 (0,02)***	<0,001
**Mortalidade por todas as causas, n (%)**					0,009
Vivo	1782 (90,34)	608 (91,44)	599 (92,85)	575 (86,65)**	
Morto	246 (9,66)	68 (8,56)	64 (7,15)	114 (13,35)**	
**Tempo de acompanhamento (meses), Média (DP)**	79,02 (2,15)	86,68 (3,19)	78,45 (2,86)	71,97 (3,69)**	0,022
**DCVA de alto risco, n (%)**					<0,001
Não	1556 (83,35)	567 (88,69)	518 (85,04)	471 (76,28)***	
Sim	472 (16,65)	109 (11.31)	145 (14,96)	218 (23,72)***	

EASIX: índice de ativação endotelial e estresse; PIR: razão pobreza-renda; IMC: índice de massa corporal; RNL: razão neutrófilo-linfócito; HEI-2015: Índice de Alimentação Saudável 2015; DCVA: doença cardiovascular aterosclerótica. ***p < 0,001, **p < 0,01, *p < 0,05.

### Associação entre o EASIX e alto risco de DCVA e mortalidade por todas as causas em pacientes com osteoartrite

**Figura 3:**
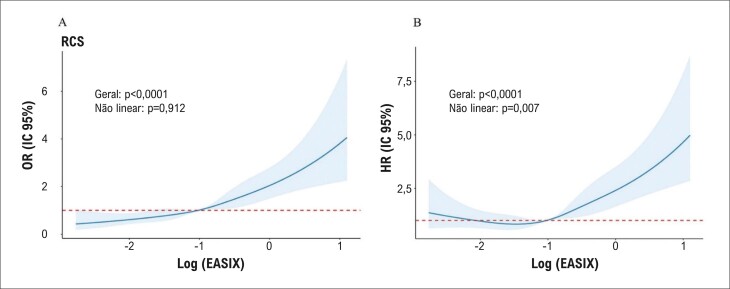
– A-B) RCS mostrando a associação entre EASIX e ORs de alto risco de DCVA em pacientes com osteoartrite.

O modelo de regressão logística univariada identificou várias variáveis de confusão associadas a um risco elevado de DCVA, incluindo nível de educação, atividade física, hábitos de consumo de álcool, estado de câncer, proporção neutrófilo-linfócito (RNL) e uso de medicamentos anti-hiperlipidêmicos ( Tabela Suplementar 3 ). No modelo bruto, um logaritmo maior (EASIX) foi associado a um risco aumentado de DCVA. Após o ajuste para potenciais fatores de confusão, a associação permaneceu significativa, com valores de logaritmo maior (EASIX) correlacionando-se com um risco maior de DCVA. Especificamente, pacientes com valores de logaritmo maior (EASIX)>-0,78 apresentaram risco significativamente maior de DCVA em comparação com aqueles com valores de log (EASIX) < -1,29( Tabela 2 ). O RCS revelou que o aumento do EASIX estava relacionado a ORs elevados de alto risco de DCVA em pacientes com OA ( Figura 3A ).

**Tabela 2:** – Associação entre EASIX e alto risco de DCVA e mortalidade por todas as causas em pacientes com osteoartrite

Variáveis	Modelo 1	Modelo 2
**Alto risco de DCVA**	**OR (IC 95%)**	**OR (IC 95%)**
Log (EASIX)	1,97 (1,57-2,48)***	1,94 (1,57-2,41)***
Log (EASIX)		
< -1,29	Ref	Ref
-1,29 - -0,78	1,38 (0,97-1,96)	1,32 (0,93-1,89)
>-0,78	2,44 (1,77-3,35)***	2,31 (1,68-3,18)***
**Mortalidade por todas as causas**	**HR (IC 95%)**	**HR (IC 95%)**
Log (EASIX)	1,84 (1,34-2,54)***	1,59 (1,14-2,23)**
Log (EASIX)		
< -1,29	Ref	Ref
-1,29 - -0,78	1,38 (0,97-1,96)	1,32 (0,93-1,89)
>-0,78	2,44 (1,77-3,35)**	2,31 (1,68-3,18)*

EASIX: índice de ativação endotelial e estresse; DCVA: doença cardiovascular aterosclerótica; OR: razão de chances; HR: razão de risco; IC: intervalo de confiança; Ref: referência. Modelo 1: Modelo bruto. Modelo 2: Ajustado para nível educacional, atividade física, estado de consumo de álcool, câncer, RNL e agentes anti-hiperlipidêmicos (para DCVA) ou relaxantes musculares e DCVA de alto risco (para mortalidade). ***p < 0,001, **p < 0,01, *p < 0,05.

**Figura 4:**
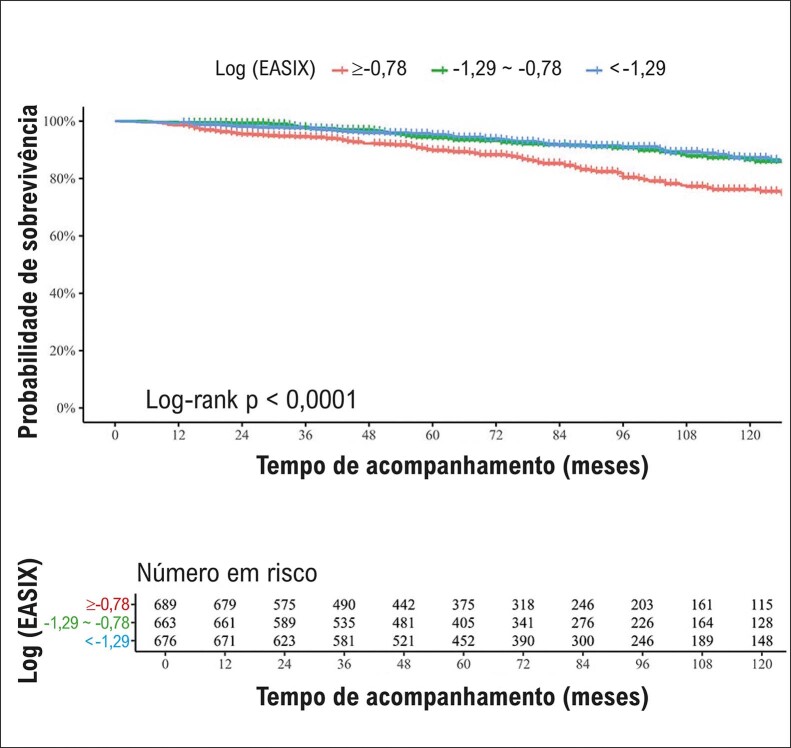
– Probabilidade de sobrevivência de participantes em diferentes grupos de logaritmo (EASIX): análise da curva de Kaplan-Meier.

Da mesma forma, o modelo de regressão univariada de Cox identificou nível de escolaridade, atividade física, estado de consumo de álcool, câncer, relaxantes musculares, RNL e DCVA de alto risco como fatores de confusão relacionados à mortalidade por todas as causas em pacientes com OA ( Tabela Suplementar 4 ). Um logaritmo aumentado (EASIX) foi associado a um maior risco de mortalidade por todas as causas. Pacientes com logaritmo (EASIX) >-0,78 apresentou um risco significativamente aumentado de mortalidade por todas as causas em comparação com aqueles com log (EASIX) < -1,29 ( Tabela 2 ). A análise RCS apoiou ainda mais uma relação positiva entre o aumento do EASIX e ORs elevados para mortalidade por todas as causas em pacientes com OA ( Figura 3B ). Além disso, a probabilidade de sobrevivência diminuiu significativamente em pacientes com log (EASIX)>-0,78 em comparação com aqueles com log (EASIX) <-1,29 ( Figura 4 ).

### Análise de subgrupos de associações do EASIX entre aqueles com risco elevado de DCVA e mortalidade geral em pacientes com osteoartrite

Análises de subgrupos foram conduzidas para explorar as associações entre o EASIX e os riscos de DCVA e mortalidade por todas as causas em diferentes subgrupos demográficos e clínicos ( Tabela 3 ). Entre os pacientes com idade ≥ 65 anos, níveis mais elevados de log (EASIX) foram associados a um risco aumentado de DCVA e mortalidade por todas as causas. Pacientes do sexo feminino com níveis mais elevados de log (EASIX) também apresentaram risco aumentado de mortalidade geral. Além disso, indivíduos negros com log (EASIX)>-0,78 apresentaram um risco significativamente elevado de mortalidade por todas as causas. As associações entre o EASIX e os riscos de DCVA e mortalidade foram consistentes em vários subgrupos, incluindo aqueles estratificados por IMC e uso de agentes anti-hiperlipidêmicos.

**Tabela 3:** – Análise de subgrupos das associações de EASIX com alto risco de DCVA e mortalidade por todas as causas em pacientes com osteoartrite

Variáveis	DCVA de alto risco		Mortalidade por todas as causas	
	Idade: <65 OR (IC 95%)	Idade: ≥65 OR (IC 95%)	Idade: <65 HR (IC 95%)	Idade: ≥65 HR (IC 95%)
Log (EASIX)	1,04 (0,58-1,86)	1,83 (1,42-2,35)***	1,43 (0,78-2,61)	1,65 (1,16-2,34)^**^
Log (EASIX)				
< -1,29	Ref	Ref	Ref	Ref
-1,29- -0,78	0,72 (0,30-1,74)	1,34 (0,89-2,03)	0,97 (0,45-2,09)	0,73 (0,39-1,36)
>-0,78	1,11 (0,52-2,38)	2,01 (1,33-3,02)***	1,55 (0,81-2,98)	1,45 (0,88-2,40)
**Variáveis**	**Sexo: Feminino**	**Sexo: Masculino**	**Sexo: Feminino**	**Sexo: Masculino**
	**OR (IC 95%)**	**OR (IC 95%)**	**HR (IC 95%)**	**HR (IC 95%)**
Log (EASIX)	1,68 (1,24-2,28) ***	1,71 (1,09-2,68)^*^	1,50 (1,01-2,22)^*^	1,35 (0,76-2,41)
Log (EASIX)				
< -1,29	Ref	Ref	Ref	Ref
-1,29 - -0,78	1,02 (0,64-1,64)	1,48 (0,76-2,85)	0,70 (0,37-1,32)	1,02 (0,40-2,64)
>-0,78	1,69 (1,05-2,72)^*^	2.16 (1.11-4.20)^*^	1,50 (0,92-2,47)	1,27 (0,56-2,92)
**Variáveis**	**Raça: Branca**	**Raça: Negra**	**Raça: Branca**	**Raça: Negra**
	**OR (IC 95%)**	**OR (IC 95%)**	**HR (IC 95%)**	**HR (IC 95%)**
Log (EASIX)	2,00 (1,53-2,62)***	2,26 (1,49-3,44)***	1,65 (1,13-2,41)**	1,82 (1,35-2,45)^***^
Log (EASIX)				
< -1,29	Ref	Ref	Ref	Ref
-1,29 - -0,78	1,36 (0,87-2,12)	0,93 (0,46-1,88)	0,84 (0,47-1,50)	2,06 (0,70-6,10)
>-0,78	2,34 (1,62-3,37)***	3,09 (1,52-6,28)**	1,51 (0,97-2,34)	4,06 (1,40-11,78)*
**Variáveis**	**IMC: <25**	**IMC: ≥25**	**IMC: <25**	**IMC: ≥25**
	**OR (IC 95%)**	**OR (IC 95%)**	**HR (IC 95%)**	**HR (IC 95%)**
Log (EASIX)	1,71 (1,15-2,52)*	2,07 (1,58-2,70)***	2,36 (1,63-3,42)***	1,46 (0,98-2,16)
Log (EASIX)				
< -1,29	Ref	Ref	Ref	Ref
-1,29 - -0,78	2,24 (1,18-4,22)*	1,14 (0,71-1,82)	1,20 (0,53-2,68)	0,77 (0,42-1,42)
>-0,78	2,21 (1,29-3,79)**	2,37 (1,61-3,49)***	3,44 (1,85-6,40)***	1,36 (0,84-2,18)
**Variáveis**	**Agentes anti-hiperlipidêmicos: Não**	**Agentes anti-hiperlipidêmicos: Sim**	**Agentes anti-hiperlipidêmicos: Não**	**Agentes anti-hiperlipidêmicos: Sim**
	**OR (IC 95%)**	**OR (IC 95%)**	**HR (IC 95%)**	**HR (IC 95%)**
Log (EASIX)	2,00 (1,51-2,66)***	1,89 (1,30-2,75)***	1,82 (1,30-2,56)***	1,24 (0,68-2,25)
Log (EASIX)				
< -1,29	Ref	Ref	Ref	Ref
-1,29 - -0,78	1,34 (0,87-2,06)	1,32 (0,72-2,44)	0,96 (0,53-1,73)	0,68 (0,24-1,89)
>-0,78	2,50 (1,51-4,14)***	2,12 (1,26-3,59)*	2,01 (1,22-3,31)**	1,02 (0,46-2,27)

EASIX: índice de ativação endotelial e estresse, DCVA: doença cardiovascular aterosclerótica, Ref: referência, OR: razão de chances, HR: razão de risco, IC: intervalo de confiança, IMC: índice de massa corporal. Quanto à DCVA de alto risco, se não estratificada, ajustada para nível de escolaridade, atividade física, consumo de álcool, câncer, RNL e agentes anti-hiperlipidêmicos. Quanto à mortalidade por todas as causas, se não estratificada, ajustada para nível de escolaridade, atividade física, consumo de álcool, câncer, relaxantes musculares, RNL e DCVA de alto risco.***p < 0,001, **p < 0,01, *p < 0,05.

## Discussão

### EASIX: Um novo biomarcador para risco cardiovascular na osteoartrite

Nosso estudo estabelece que o EASIX prediz de forma independente a DCVA e a mortalidade por todas as causas em pacientes com OA. Essa associação provavelmente decorre da capacidade do EASIX de quantificar a disfunção endotelial sistêmica, um processo impulsionado pela inflamação crônica e pela desregulação metabólica inerentes à OA. Os componentes do EASIX—lactato desidrogenase (LDH).^19^ creatinina^20^e contagem de plaquetas—refletem coletivamente os principais mecanismos patológicos:^21 - 23^ A elevação da LDH reflete a sobrecarga glicolítica nas articulações inflamadas, os níveis de creatinina sinalizam a perda muscular associada à imobilidade articular e a depleção de plaquetas pode indicar lesão microvascular.^24 , 25^ Ao integrar esses marcadores, o EASIX fornece uma visão holística do estresse vascular que complementa as ferramentas tradicionais de avaliação de risco cardiovascular.

### Tradução clínica em uma sociedade em envelhecimento

A simplicidade do EASIX, derivada de parâmetros laboratoriais de rotina, o posiciona como uma ferramenta pragmática para estratificação de risco na gestão do AO.^26^À medida que a população global envelhece, a dupla carga da OA e das DCV exige soluções escaláveis. O EASIX oferece vantagens únicas: é econômico e não requer testes especializados,^27^ e identifica pacientes de alto risco (por exemplo, log (EASIX) >-0,78) que podem se beneficiar de intervenções precoces, como estatinas, terapias antiinflamatórias ou cuidados multidisciplinares.^28^ Para adultos mais velhos (≥65 anos), a triagem obrigatória do EASIX pode reduzir a morbidade, permitindo encaminhamentos oportunos para cardiologia ou programas preventivos.^9 , 29^ Essa abordagem está alinhada às prioridades globais de saúde para otimizar a alocação de recursos em populações idosas.

### Limitações e direções futuras

Embora nossos resultados destaquem o valor prognóstico do EASIX, suas limitações incluem a falta de dados sobre a gravidade da OA no NHANES e potenciais fatores de confusão não mensurados. Estudos futuros devem validar os limiares do EASIX em diversas coortes, incluindo OA em estágio inicial e populações sub-representadas, e investigar sua utilidade na orientação de terapias direcionadas (por exemplo, inibidores da glicólise) ou políticas de saúde pública.

## Conclusões

Este estudo estabelece o EASIX como um novo biomarcador para identificar pacientes com OA com risco elevado de DCVA e mortalidade por todas as causas. Valores mais altos de EASIX se correlacionam com aumento do estresse vascular e inflamação sistêmica, refletindo a interação entre a fisiopatologia relacionada à OA e a disfunção endotelial. A simplicidade do EASIX — calculado a partir de parâmetros laboratoriais de rotina — o posiciona como uma ferramenta prática para estratificação de risco na prática clínica, particularmente para populações em envelhecimento onde a OA e comorbidades cardiovasculares são prevalentes.

### Implicações clínicas e de saúde pública

A integração do EASIX às avaliações de rotina de pacientes com OA pode aprimorar a identificação precoce de indivíduos de alto risco, possibilitando intervenções direcionadas, como a intensificação da prevenção cardiovascular ou o atendimento multidisciplinar. Sua relação custo-efetividade e a dependência de testes amplamente disponíveis tornam o EASIX particularmente valioso em ambientes com recursos limitados, alinhando-se às prioridades globais para abordar a dupla carga das doenças musculoesqueléticas e cardiovasculares crônicas.

### Direções futuras

É essencial uma validação mais aprofundada dos limiares EASIX em diversas populações e subtipos de OA. A pesquisa também deve explorar se as terapias guiadas pelo EASIX (por exemplo, agentes anti-inflamatórios ou protetores endoteliais) melhoram os resultados e como este biomarcador pode subsidiar estratégias de saúde pública para sociedades em envelhecimento.

## * Material suplementar

Para informação adicional da Tabela Suplementar 1, por favor, clique aqui.



Para informação adicional da Tabela Suplementar 2, por favor, clique aqui.



Para informação adicional da Tabela Suplementar 3, por favor, clique aqui.



Para informação adicional da Tabela Suplementar 4, por favor, clique aqui.


