# Supplementary material for: Associação da Ativação Endotelial e do Índice de Estresse com Risco de Doença Cardiovascular e Mortalidade por Todas as Causas em Pacientes com Osteoartrite
Source: Arq Bras Cardiol. 2025 Jul 10;122(7):e20250012. [Article in Portuguese] doi: 10.36660/abc.20250012 (PMC12296238; doi:10.36660/abc.20250012)
Supplement: Supplementary file 1 [file 2025-0012_AO_Supplementary_Table_1.pdf]

Supplementary Table 1 The missing value status of variables

Variables	n	%
PIR	150	7.40
BMI	24	1.18
Vitamin D	36	1.78
HEI	86	4.24
Education level	1	0.05
NLR	1	0.05

PIR: poverty-to-income ratio, BMI: body mass index, HEI-2015: Healthy Eating Index 2015, NLR: neutrophil-to-lymphocyte ratio
