# Supplementary material for: Associação da Ativação Endotelial e do Índice de Estresse com Risco de Doença Cardiovascular e Mortalidade por Todas as Causas em Pacientes com Osteoartrite
Source: Arq Bras Cardiol. 2025 Jul 10;122(7):e20250012. [Article in Portuguese] doi: 10.36660/abc.20250012 (PMC12296238; doi:10.36660/abc.20250012)
Supplement: Supplementary file 2 [file 2025-0012_AO_Supplementary_Table_2.pdf]

Supplementary Table 2 Sensitivity analysis of data between before and after multiple imputation

Variables	Before imputation	After imputation	Statistics	*P*
PIR			χ^2=3.223	0.073
<1.3	443 (13.74)	483 (14.27)		
≥1.3	1435 (86.26)	1545 (85.73)		
BMI			χ^2=0.005	0.943
<25	386 (20.09)	389 (20.08)		
≥25	1618 (79.91)	1639 (79.92)		
Vitamin D			χ^2=0.602	0.438
<75	895 (35.34)	909 (35.47)		
≥75	1097 (64.66)	1119 (64.53)		
HEI	53.30 (0.48)	53.28 (0.47)	t=0.300	0.764
Education			χ^2=0.985	0.321
Under high school	350 (10.07)	350 (10.07)		
High school and above	1677 (89.93)	1678 (89.93)		
NLR, Mean (S.E)	2.29 (0.04)	2.29 (0.04)	t=-1.000	0.321

PIR: poverty-to-income ratio, BMI: body mass index, HEI-2015: Healthy Eating Index 2015, NLR: neutrophil-to-lymphocyte ratio
