# Supplementary material for: Associação da Ativação Endotelial e do Índice de Estresse com Risco de Doença Cardiovascular e Mortalidade por Todas as Causas em Pacientes com Osteoartrite
Source: Arq Bras Cardiol. 2025 Jul 10;122(7):e20250012. [Article in Portuguese] doi: 10.36660/abc.20250012 (PMC12296238; doi:10.36660/abc.20250012)
Supplement: Supplementary file 3 [file 2025-0012_AO_Supplementary_Table_3.pdf]

Supplementary Table 3 Screening of the confounding factors associated with high risk of ASCAD

Variables	OR (95%CI)	P
Education		
Under high school	Ref	
High school and above	0.59 (0.43-0.80)	**0.001**
PIR		
<1.3	Ref	
≥1.3	0.88 (0.63-1.23)	0.444
Physical activity (MET × min/week)		
<750	Ref	
≥750	0.66 (0.51-0.84)	**<0.001**
Drinking status		
No	Ref	
Yes	0.62 (0.45-0.83)	**0.002**
Unknown	0.83 (0.55-1.26)	0.374
Depression		
No	Ref	
Yes	0.84 (0.49-1.43)	0.513
Cancer		
No	Ref	
Yes	2.40 (1.69-3.40)	**<0.001**
Anti-hyperlipidemic agents		
No	Ref	
Yes	2.70 (2.09-3.48)	**<0.001**
Adrenal cortical steroids		
No	Ref	
Yes	2.12 (0.89-5.05)	0.091
Analgesics		
No	Ref	
Yes	0.86 (0.60-1.22)	0.392
Muscle relaxants		
No	Ref	
Yes	0.82 (0.47-1.42)	0.464
BMI (kg/m^2)		
<25	Ref	
≥25	1.24 (0.91-1.68)	0.165
Vitamin D (nmol/L)		
<75	Ref	
≥75	0.91 (0.68-1.22)	0.525
NLR	1.22 (1.10-1.35)	**<0.001**
HEI 2015	1.00 (0.99-1.01)	0.741

Ref: reference, OR: odds ratio, CI: confidence interval, PIR: poverty-to-income ratio, BMI: body mass index, NLR: neutrophil-to-lymphocyte ratio, HEI-2015: Healthy Eating Index 2015, ASCVD: atherosclerotic cardiovascular disease
