# Supplementary material for: Associação da Ativação Endotelial e do Índice de Estresse com Risco de Doença Cardiovascular e Mortalidade por Todas as Causas em Pacientes com Osteoartrite
Source: Arq Bras Cardiol. 2025 Jul 10;122(7):e20250012. [Article in Portuguese] doi: 10.36660/abc.20250012 (PMC12296238; doi:10.36660/abc.20250012)
Supplement: Supplementary file 4 [file 2025-0012_AO_Supplementary_Table_4.pdf]

Supplementary Table 4 Screening of the confounding factors associated with all-cause mortality

Variables	HR	P
Education		
Under high school	Ref	
High school and above	0.62 (0.41-0.94)	**0.026**
PIR		
<1.3	Ref	
≥1.3	0.78 (0.56-1.08)	0.134
Physical activity (MET × min/week)		
<750	Ref	
≥750	0.63 (0.44-0.91)	**0.013**
Drinking status		
No	Ref	
Yes	1.56 (1.07-2.26)	**0.021**
Unknown	1.67 (0.75-3.75)	0.209
Depression		
No	Ref	
Yes	1.18 (0.73-1.91)	0.506
Cancer		
No	Ref	
Yes	1.60 (1.06-2.42)	**0.025**
Anti-hyperlipidemic agents		
No	Ref	
Yes	1.22 (0.88-1.69)	0.228
Adrenal cortical steroids		
No	Ref	
Yes	1.66 (0.65-4.26)	0.290
Analgesics		
No	Ref	
Yes	1.30 (0.90-1.87)	0.164
Muscle relaxants		
No	Ref	
Yes	2.03 (1.24-3.30)	**0.005**
BMI (kg/m^2)		
<25	Ref	
≥25	0.97 (0.67-1.40)	0.857
Vitamin D (nmol/L)		
<75	Ref	
≥75	1.02 (0.75-1.40)	0.890
NLR	1.13 (1.03-1.24)	**0.008**
HEI 2015	0.99 (0.98-1.00)	0.055

Variables	HR	P
High risk ASCVD		
No	Ref	
Yes	3.31 (2.20-4.98)	**<0.001**

Ref: reference, HR: hazard ratio, CI: confidence interval, PIR: poverty-to-income ratio, BMI: body mass index, NLR: neutrophil-to-lymphocyte ratio, HEI-2015: Healthy Eating Index 2015, ASCVD: atherosclerotic cardiovascular disease
