# Supplementary material for: Associação da Ativação Endotelial e do Índice de Estresse com Risco de Doença Cardiovascular e Mortalidade por Todas as Causas em Pacientes com Osteoartrite
Source: Arq Bras Cardiol. 2025 Jul 10;122(7):e20250012. [Article in Portuguese] doi: 10.36660/abc.20250012 (PMC12296238; doi:10.36660/abc.20250012)
Supplement: Translation

# Translation: Association of Endothelial Activation and Stress Index with Risk of Cardiovascular Disease and All-cause Mortality in Patients with Osteoarthritis

**DOI:** 10.36660/abc.20250012i

**Authors:** Ruoyan Li, Chaoqun Feng, Kexin Lin, Nan Wang, Xiaohong Fan

**Affiliations:** 1 China Hospital of Chengdu University of Traditional Chinese Medicine – Department of Orthopedics, Chengdu, Sichuan – China

**Keywords:** Osteoarthritis, Cardiovascular Diseases, Mortality

## Abstract

**Background:**

Osteoarthritis is a prevalent type of arthritis characterized by chronic degenerative changes in the musculoskeletal system, which can result in joint damage and chronic pain.

**Objective:**

This study was to investigate the associations between the endothelial activation and stress index (EASIX) and the increased risk of atherosclerotic cardiovascular disease (ASCVD) and all-cause mortality among patients diagnosed with osteoarthritis.

**Methods:**

The cohort study encompassed 2028 individuals aged 40-79 years with osteoarthritis, utilizing data from the National Health and Nutrition Examination Surveys (NHANES) database spanning the years 2007 to 2018. The univariate weighted logistic regression model and weighted Cox model were respectively established to screen possible confounders. A significance level of p < 0.05 was adopted for all statistical analyses.

**Results:**

The study revealed an elevated risk of ASCVD in correlation with an increased log (EASIX) (Odds Ratio: 1.94, with 95% Confidence Interval:1.57-2.41). When compared to individuals with log (EASIX)< -1.29, those with a log (EASIX) > -0.78 demonstrated a heightened risk of ASCVD (Odds Ratio: 2.31, with 95% Confidence Interval:1.68-3.18). A higher log (EASIX) value was also linked to an increased risk of mortality from all causes (Hazard Ratio: 1.59, with 95% Confidence Interval:1.14 -2.23). Among individuals diagnosed with osteoarthritis, those exhibiting log (EASIX)> -0.78 faced a greater risk of dying from any cause, as compared to patients with log (EASIX) <-1.29.

**Conclusion:**

The presence of a high EASIX index was linked to an increased risk of ASCVD and all-cause mortality among patients with osteoarthritis.

## Introduction

Osteoarthritis (OA) is a prevalent degenerative joint disease associated with chronic pain, functional decline, and reduced quality of life.^1^While current treatments focus on symptom management, they do not halt disease progression or prevent joint damage.^2^Notably, OA is increasingly recognized as a risk factor for cardiovascular disease (CVD),^3 , 4^which remains a leading cause of global mortality.^5^Identifying OA patients at elevated CVD risk is critical for improving prognosis and reducing disease burden.

Endothelial dysfunction, a key mechanism in atherosclerosis and CVD, may underlie the increased CVD risk in OA patients.^6 , 7^Chronic inflammation in OA leads to the release of inflammatory mediators into the circulation, promoting endothelial damage and atherosclerosis progression.^8^

The endothelial activation and stress index (EASIX) has recently emerged as a novel marker of endothelial injury,^9^suggesting its potential utility in assessing CVD risk in OA patients.

The American College of Cardiology and American Heart Association’s 10-year atherosclerotic cardiovascular disease (ASCVD) risk score is widely used for CVD risk assessment.^10 , 11^In this study, we aimed to evaluate the association between EASIX and high ASCVD risk, as well as overall mortality, in OA patients using data from the National Health and Nutrition Examination Surveys (NHANES). Subgroup analyses were conducted to explore the impact of age, gender, ethnicity, body mass index (BMI), and anti-hyperlipidemic medication use ( Central Illustration ).

## Materials and methods

### Study design and population

This cohort study included 2824 patients with OA aged 40-79 years in the NHANES database from 2007 to 2018. The NHANES program is a comprehensive, continuous study being conducted with the objective of mirroring the comprehensive demographic profile of the non-institutionalized civilian population across the United States by employing a sophisticated, multi-stage sampling method with probabilistic clustering in its research design, which provides comprehensive insights into diverse health parameters, encompassing lifestyle choices, physical fitness levels, dietary habits, and mental well-being.^12^In our study, participants diagnosed with OA, aged 40-79 years old, with complete data for EASIX calculation, with assessment of 10-year ASCVD, and with complete survival data in the NHANES 2007-2018 were included. Participants with self-reported CVD were excluded ( Central Illustration ).

### Potential covariates and definitions

Education was categorized as either below the level of a high school diploma or possessing at least a high school diploma, poverty-to-income ratio (PIR), physical activity (MET × min/week), BMI (kg/m^2^), and vitamin D (nmol/L) were respectively dichotomized as <1.3 or ≥1.3, <750 or ≥750, <25 or ≥ 25 and <75 or ≥75. Drinking status, depression, cancer, anti-hyperlipidemic agents, adrenal cortical steroids, analgesics, and muscle relaxants were dichotomized as yes or no. The neutrophil-to-lymphocyte ratio (NLR) and the Healthy Eating Index 2015 (HEI-2015) were utilized as continuous variables.

The face-to-face interview conducted at the MEC utilized the Patient Health Questionnaire-9 (PHQ-9)^13^to assess depressive symptoms experienced within the preceding two weeks. Respondents rated the frequency of nine depressive symptoms (e.g., anhedonia, depressed mood, sleep disturbances) on a 0-3 scale during an assessment. Total scores, ranging from 0 to 27, with scores of 10 or higher indicating clinically significant depressive symptoms.^14^Physical activity was converted to energy consumption, and the energy consumption (MET×min) = suggested metabolic equivalent (MET) × corresponding activities of sports time (min). Vitamin D ≥75 nmol/L was defined as adequate, < 75 nmol/L as inadequate.^15^The HEI-2015 focuses on nine food groups for balanced consumption (such as fruits, vegetables, whole grains, dairy, and proteins from various sources) while also emphasizing a healthy unsaturated-to-saturated fat ratio. Four components (refined grains, sodium, added sugars, and saturated fats) should be consumed in moderation. Each component’s intake is standardized using the density method with a 4184 kJ reference. Components are assigned a score ranging from 0 to 5 or 0 to 10. By summing up these component scores for each respondent with a maximum possible score of 100 points, an overall score can be calculated. The population ratio method is appropriately utilized for calculating mean scores.^16 , 17^

#### Main variable

EASIX score was the main variable. The formula was used to calculate EASIX: serum lactate dehydrogenase (LDH) level (U/L) × creatinine level (mg/dL)/platelet count (10^9^/L). Since EASIX exhibited a skewed distribution, it underwent a log^2^transformation prior to analysis.^18^ Figure 1 displays the EASIX data both before and after applying the log^2^transformation.

**Figure 1:** – The distribution of EASIX data before and after log2 transformed.

#### Outcome variables

The high CVD risk was the primary outcome, which was evaluated by the 10-year ASCVD risk score based on factors such as age, gender, and ethnicity. Another example would be cholesterol levels, blood pressure, and medication use.^10^The criteria for assessing the 10-year risk of CVD categorize individuals into four risk levels: low risk (<5%), borderline risk (5% to 7.5%), intermediate risk (7.5% to <20%), and high risk (≥20%). In this study, we aimed to explore the high CVD risk of patients.

The secondary outcome focused on the all-cause mortality rate among OA patients, evaluated by linking their death certificate records with the National Death Index (NDI). All-cause mortality refers to the total number of deaths that occurred throughout the follow-up period, extending up to December 2019.

## Statistical analysis

The normality of the data was assessed using the Shapiro-Wilk test, and variables that did not meet the assumption of normality were appropriately transformed. For comparisons among multiple groups, one-way Analysis of Variance (ANOVA) was used, followed by Tukey’s post hoc test to identify specific group differences when ANOVA results were significant. Categorical data were presented as frequencies and percentages [n (%)], and comparisons among groups were performed using the chi-square test or Fisher’s exact probability method, as appropriate. The status of missing values for the variables is presented in Supplementary Table 1 , and multiple imputation was applied. Comparisons of data between before and after multiple imputations were exhibited in Supplementary Table 2 . Univariate weighted logistic regression model, and weighted Cox model were respectively established to screen possible confounders. Univariate and multivariate weighted logistic regression, weighted model, and weighted Cox model were respectively applied to analyze the associations of EASIX with high-risk ASCVD and all-cause mortality in OA patients. The subgroup analysis was categorized based on age, gender, ethnicity, BMI, and anti-hyperlipidemic agents. The OR and HR with their respective 95% CI were calculated. The statistical analysis was carried out with SAS 9.4, with statistical significance set at a p-value of less than 0.05.

## Results

### Characteristics of Participants Based on Different EASIX Scores

In total, 2824 patients with OA aged 40-79 years in the NHANES database from 2007 to 2018 were identified. Individuals who had previously experienced CVD were not included in the study (n=554). Participants without complete data for EASIX calculation (n=180), without complete survival data (n=4), or the assessment of 10-year ASCVD (n=58) were excluded. At last, 2028 participants were included, and among them, 472 participants had a high risk of ASCVD. The screening process of participants is shown in Figure 2 .

**Figure 2:** – The screening process of the participants in this study. CVD: cardiovascular disease; ASCVD: atherosclerotic cardiovascular disease.

The characteristics of participants across different log (EASIX) groups are presented in Table 1 . Significant differences were observed in various demographic and clinical variables, including age, gender, race, physical activity, smoking status, drinking status, and comorbidities such as diabetes, hypertension, and dyslipidemia. Additionally, differences in laboratory parameters such as lactate dehydrogenase (LDH) levels, platelet counts, and creatinine levels were noted among the groups. The mortality rates and the proportion of participants with a high risk of ASCVD also varied significantly across the log (EASIX) groups.

**Table 1:** – The characteristics of participants with different EASIX levels

Variables	Total (n=2028)	Log (EASIX)			p
		< -1.29 (n=676)	-1.29- -0.78 (n=663)	≥-0.78 (n=689)	
**Age (years), Mean (SD)**	60.88 (0.31)	59.06 (0.47)	60.91 (0.49)	62.65 (0.52)***	<0.001
**Gender, n (%)**					<0.001
Female	1337 (65.43)	576 (87.03)	452 (64.51)	309 (44.83)***	
Male	691 (34.57)	100 (12.97)	211 (35.49)	380 (55.17)***	
**Race, n (%)**					0.008
White	1157 (82.90)	370 (81.67)	382 (82.42)	405 (84.61)**	
Black	347 (6.42)	93 (5.60)	115 (6.30)	139 (7.37)**	
Others	524 (10.68)	213 (12.73)	166 (11.28)	145 (8.02)**	
**Education, n (%)**					0.244
Under high school	350 (10.07)	131 (11.69)	106 (9.07)	113 (9.49)	
High school and above	1678 (89.93)	545 (88.31)	557 (90.93)	576 (90.51)	
**PIR, n (%)**					0.355
<1.3	483 (14.27)	166 (14.16)	170 (15.68)	147 (12.90)	
≥1.3	1545 (85.73)	510 (85.84)	493 (84.32)	542 (87.10)	
**Physical activity (MET × min/week), n (%)**					0.015
<750	1060 (46.91)	373 (51.82)	361 (47.99)	326 (40.90)**	
≥750	968 (53.09)	303 (48.18)	302 (52.01)	363 (59.10)**	
**Smoking status, n (%)**					0.003
No	1697 (85.12)	535 (81.05)	552 (84.95)	610 (89.37)**	
Yes	331 (14.88)	141 (18.95)	111 (15.05)	79 (10.63)**	
**Drinking status, n (%)**					<0.001
No	456 (17.17)	190 (22.41)	148 (15.75)	118 (13.42)***	
Yes	1176 (61.84)	391 (62.63)	388 (63.20)	397 (59.64)***	
Unknown	396 (20.99)	95 (14.96)	127 (21.05)	174 (26.94)***	
**Diabetes, n (%)**					0.166
No	1528 (80.67)	498 (78.95)	514 (83.24)	516 (79.73)	
Yes	500 (19.33)	178 (21.05)	149 (16.76)	173 (20.27)	
**Hypertension**					0.763
No	458 (26.03)	176 (25.08)	154 (27.28)	128 (25.70)	
Yes	1570 (73.97)	500 (74.92)	509 (72.72)	561 (74.30)	
**Dyslipidemia, n (%)**					0.044
No	355 (17.67)	110 (16.27)	136 (21.40)	109 (15.20)**	
Yes	1673 (82.33)	566 (83.73)	527 (78.60)	580 (84.80)**	
**Depression, n (%)**					0.435
No	1800 (90.86)	586 (90.01)	589 (90.26)	625 (92.33)	
Yes	228 (9.14)	90 (9.99)	74 (9.74)	64 (7.67)	
**Cancer, n (%)**					0.018
No	1665 (79.58)	569 (83.20)	552 (79.97)	544 (75.58)**	
Yes	363 (20.42)	107 (16.80)	111 (20.03)	145 (24.42)**	
**Anti-hyperlipidemic agents**					0.002
No	1352 (67.73)	484 (74.03)	447 (66.32)	421 (62.90)***	
Yes	676 (32.27)	192 (25.97)	216 (33.68)	268 (37.10)***	
**Adrenal cortical steroids, n (%)**					0.739
No	1973 (98.06)	659 (98.40)	648 (97.74)	666 (98.05)	
Yes	55 (1.94)	17 (1.60)	15 (2.26)	23 (1.95)	
**Analgesics, n (%)**					0.630
No	1412 (70.43)	458 (68.85)	454 (70.70)	500 (71.73)	
Yes	616 (29.57)	218 (31.15)	209 (29.30)	189 (28.27)	
**Muscle relaxants, n (%)**					0.528
No	1886 (93.26)	624 (92.29)	615 (93.23)	647 (94.25)	
Yes	142 (6.74)	52 (7.71)	48 (6.77)	42 (5.75)	
**BMI (kg/m^**2**^), n (%)**					0.009
<25	389 (20.08)	144 (23.63)	130 (21.07)	115 (15.51)**	
≥25	1639 (79.92)	532 (76.37)	533 (78.93)	574 (84.49)**	
**Vitamin D (nmol/L), n (%)**					0.293
<75	909 (35.47)	303 (36.09)	315 (37.90)	291 (32.34)	
≥75	1119 (64.53)	373 (63.91)	348 (62.10)	398 (67.66)	
**NLR, Mean (SD)**	2.29 (0.04)	2.24 (0.05)	2.25 (0.09)	2.38 (0.07)	0.280
**HEI 2015, Mean (SD)**	53.28 (0.47)	53.01 (0.75)	53.71 (0.80)	53.10 (0.71)	0.772
**Lactate dehydrogenase LDH (U/L), Mean (SD)**	140.04 (0.98)	125.40 (1.40)	139.42 (1.42)	155.27 (1.64)***	<0.001
**Platelet count (1000 cells/uL), Mean (SD)**	242.65 (2.30)	289.09 (3.27)	237.73 (2.54)	201.41 (2.08)***	<0.001
**Creatinine (mg/dL), Mean (SD)**	0.87 (0.01)	0.72 (0.01)	0.85 (0.01)	1.05 (0.02)***	<0.001
**EASIX, Mean (SD)**	0.54 (0.01)	0.32 (0.00)	0.50 (0.00)	0.82 (0.02)***	<0.001
**Log (EASIX), Mean (SD)**	-1.02 (0.02)	-1.69 (0.02)	-1.02 (0.01)	-0.35 (0.02)***	<0.001
**All-cause mortality, n (%)**					0.009
Alive	1782 (90.34)	608 (91.44)	599 (92.85)	575 (86.65)**	
Dead	246 (9.66)	68 (8.56)	64 (7.15)	114 (13.35)**	
**Follow-up time (months), Mean (SD)**	79.02 (2.15)	86.68 (3.19)	78.45 (2.86)	71.97 (3.69)**	0.022
**High risk ASCVD, n (%)**					<0.001
No	1556 (83.35)	567 (88.69)	518 (85.04)	471 (76.28)***	
Yes	472 (16.65)	109 (11.31)	145 (14.96)	218 (23.72)***	

EASIX: endothelial activation and stress index, PIR: poverty-to-income ratio, BMI: body mass index, NLR: neutrophil-to-lymphocyte ratio, HEI-2015: Healthy Eating Index 2015, ASCVD: atherosclerotic cardiovascular disease. ***p < 0.001, **p < 0.01, *p < 0.05.

### Association between EASIX and High Risk of ASCVD and All-Cause Mortality in Patients with Osteoarthritis

The univariate logistic regression model identified several confounding variables associated with an elevated risk of ASCVD, including education level, physical activity, drinking habits, cancer status, neutrophil-to-lymphocyte ratio (NLR), and the use of anti-hyperlipidemic medications ( Supplementary Table 3 ). In the crude model, a higher log (EASIX) was associated with an increased risk of ASCVD. After adjusting for potential confounders, the association remained significant, with higher log (EASIX) values correlating with a greater risk of ASCVD. Specifically, patients with log (EASIX) values > -0.78 had a significantly higher risk of ASCVD compared to those with log (EASIX) values < -1.29 ( Table 2 ). RCS revealed that increased EASIX was related to elevated ORs of high risk of ASCVD in patients with OA ( Figure 3A ).

**Figure 3:** – A-B RCS showing the association between EASIX and ORs of high risk of ASCVD in patients with osteoarthritis.

Similarly, the univariate Cox regression model identified education level, physical activity, drinking status, cancer, muscle relaxants, NLR, and high-risk ASCVD as confounding factors related to all-cause mortality in OA patients ( Supplementary Table 4 ). An increased log (EASIX) was associated with a higher risk of all-cause mortality. Patients with log (EASIX) >-0.78 exhibited a significantly increased risk of all-cause mortality compared to those with log (EASIX) < -1.29 ( Table 2 ). RCS analysis further supported a positive relationship between increased EASIX and heightened ORs for all-cause mortality in OA patients ( Figure 3B ). Additionally, survival probability was significantly decreased in patients with log(EASIX) >-0.78 compared to those with log(EASIX) <-1.29 (Figure 5).

**Figure 4:** – Survival Probability of Participants Across Different Log (EASIX) Groups: Kaplan-Meier Curve Analysis.

### Subgroup analysis of associations of EASIX among those with elevated risk of ASCVD and overall mortality in patients with osteoarthritis

Subgroup analyses were conducted to explore the associations between EASIX and the risks of ASCVD and all-cause mortality across different demographic and clinical subgroups ( Table 3 ). Among patients aged ≥65 years, higher log (EASIX) levels were associated with an increased risk of both ASCVD and all-cause mortality. Female patients with higher log (EASIX) levels also exhibited an increased risk of overall mortality. Additionally, Black individuals with log (EASIX) > -0.78 had a significantly elevated risk of all-cause mortality. The associations between EASIX and the risks of ASCVD and mortality were consistent across various subgroups, including those stratified by BMI and the use of anti-hyperlipidemic agents.

**Table 3:** – Subgroup analysis of associations of EASIX with high risk of ASCVD and all-cause mortality in patients with osteoarthritis

Variables	High-risk ASCVD		All-cause mortality	
	Age: <65 OR (95%CI)	Age: ≥65 OR (95%CI)	Age: <65 HR (95%CI)	Age: ≥65 HR (95%CI)
Log (EASIX)	1.04 (0.58-1.86)	1.83 (1.42-2.35)***	1.43 (0.78-2.61)	1.65 (1.16-2.34)^**^
Log (EASIX)				
< -1.29	Ref	Ref	Ref	Ref
-1.29- -0.78	0.72 (0.30-1.74)	1.34 (0.89-2.03)	0.97 (0.45-2.09)	0.73 (0.39-1.36)
>-0.78	1.11 (0.52-2.38)	2.01 (1.33-3.02)***	1.55 (0.81-2.98)	1.45 (0.88-2.40)
**Variables**	**Gender: Female**	**Gender: Male**	**Gender: Female**	**Gender: Male**
	**OR (95%CI)**	**OR (95%CI)**	**HR (95%CI)**	**HR (95%CI)**
Log (EASIX)	1.68 (1.24-2.28) ***	1.71 (1.09-2.68)^*^	1.50 (1.01-2.22)^*^	1.35 (0.76-2.41)
Log (EASIX)				
< -1.29	Ref	Ref	Ref	Ref
-1.29 - -0.78	1.02 (0.64-1.64)	1.48 (0.76-2.85)	0.70 (0.37-1.32)	1.02 (0.40-2.64)
>-0.78	1.69 (1.05-2.72)^*^	2.16 (1.11-4.20)^*^	1.50 (0.92-2.47)	1.27 (0.56-2.92)
**Variables**	**Race: White**	**Race: Black**	**Race: White**	**Race: Black**
	**OR (95%CI)**	**OR (95%CI)**	**HR (95%CI)**	**HR (95%CI)**
Log (EASIX)	2.00 (1.53-2.62)***	2.26 (1.49-3.44)***	1.65 (1.13-2.41)**	1.82 (1.35-2.45)^***^
Log (EASIX)				
< -1.29	Ref	Ref	Ref	Ref
-1.29 - -0.78	1.36 (0.87-2.12)	0.93 (0.46-1.88)	0.84 (0.47-1.50)	2.06 (0.70-6.10)
>-0.78	2.34 (1.62-3.37)***	3.09 (1.52-6.28)**	1.51 (0.97-2.34)	4.06 (1.40-11.78)*
**Variables**	**BMI: <25**	**BMI: ≥25**	**BMI: <25**	**BMI: ≥25**
	**OR (95%CI)**	**OR (95%CI)**	**HR (95%CI)**	**HR (95%CI)**
Log (EASIX)	1.71 (1.15-2.52)*	2.07 (1.58-2.70)***	2.36 (1.63-3.42)***	1.46 (0.98-2.16)
Log (EASIX)				
< -1.29	Ref	Ref	Ref	Ref
-1.29 - -0.78	2.24 (1.18-4.22)*	1.14 (0.71-1.82)	1.20 (0.53-2.68)	0.77 (0.42-1.42)
>-0.78	2.21 (1.29-3.79)**	2.37 (1.61-3.49)***	3.44 (1.85-6.40)***	1.36 (0.84-2.18)
**Variables**	**Anti-hyperlipidemic agents: No**	**Anti-hyperlipidemic agents: Yes**	**Anti-hyperlipidemic agents: No**	**Anti-hyperlipidemic agents: Yes**
	**OR (95%CI)**	**OR (95%CI)**	**HR (95%CI)**	**HR (95%CI)**
Log (EASIX)	2.00 (1.51-2.66)***	1.89 (1.30-2.75)***	1.82 (1.30-2.56)***	1.24 (0.68-2.25)
Log (EASIX)				
< -1.29	Ref	Ref	Ref	Ref
-1.29 - -0.78	1.34 (0.87-2.06)	1.32 (0.72-2.44)	0.96 (0.53-1.73)	0.68 (0.24-1.89)
>-0.78	2.50 (1.51-4.14)***	2.12 (1.26-3.59)*	2.01 (1.22-3.31)**	1.02 (0.46-2.27)

EASIX: endothelial activation and stress index, ASCVD: atherosclerotic cardiovascular disease, Ref: reference, OR: odds ratio, HR: hazard ratio, CI: confidence interval, BMI: body mass index. As for high-risk ASCVD, if not stratified, adjusted for education level, physical activity, drinking status, cancer, NLR, and anti-hyperlipidemic agents. As for all-cause mortality, if not stratified, adjusted for education level, physical activity, drinking status, cancer, muscle relaxants, NLR, and high-risk ASCVD. ***p < 0.001, **p < 0.01, *p < 0.05.

## Discussion

### EASIX: A Novel Biomarker for Cardiovascular Risk in Osteoarthritis

Our study establishes that elevated EASIX independently predicts ASCVD and all-cause mortality in patients with OA. This association likely stems from EASIX’s ability to quantify systemic endothelial dysfunction, a process driven by chronic inflammation and metabolic dysregulation inherent to OA. The components of EASIX—lactate dehydrogenase (LDH).^19^creatinine^20^and platelet count—collectively reflect key pathological mechanisms:^21 - 23^LDH elevation mirrors glycolytic overload in inflamed joints, creatinine levels signal muscle wasting linked to joint immobility, and platelet depletion may indicate microvascular injury.^24 , 25^By integrating these markers, EASIX provides a holistic view of vascular stress that complements traditional cardiovascular risk assessment tools.

#### Clinical translation in an aging society

The simplicity of EASIX, derived from routine laboratory parameters, positions it as a pragmatic tool for risk stratification in OA management.^26^As global populations age, the dual burden of OA and CVD demands scalable solutions. EASIX offers unique advantages: it is cost-effective, requires no specialized testing,^27^and identifies high-risk patients (e.g., log(EASIX) >-0.78) who may benefit from early interventions such as statins, anti-inflammatory therapies, or multidisciplinary care.^28^For older adults (≥65 years), mandatory EASIX screening could reduce morbidity by enabling timely referrals to cardiology or preventive programs.^9 , 29^This approach aligns with global health priorities to optimize resource allocation in aging populations.

## Limitations and future directions

While our findings highlight EASIX’s prognostic value, limitations include the lack of OA severity data in NHANES and potential unmeasured confounders. Future studies should validate EASIX thresholds in diverse cohorts, including early-stage OA and underrepresented populations, and investigate its utility in guiding targeted therapies (e.g., glycolysis inhibitors) or public health policies.

## Conclusions

This study establishes the EASIX as a novel biomarker for identifying OA patients at elevated risk of ASCVD and all-cause mortality. Higher EASIX values correlate with increased vascular stress and systemic inflammation, reflecting the interplay between OA-related pathophysiology and endothelial dysfunction. The simplicity of EASIX—calculated from routine laboratory parameters—positions it as a practical tool for risk stratification in clinical practice, particularly for aging populations where OA and cardiovascular comorbidities are prevalent.

### Clinical and public health implications

Integrating EASIX into routine assessments of OA patients could enhance the early identification of high-risk individuals, enabling targeted interventions such as intensified cardiovascular prevention or multidisciplinary care. Its cost-effectiveness and reliance on widely available tests make EASIX particularly valuable in resource-limited settings, aligning with global priorities to address the dual burden of chronic musculoskeletal and CVDs.

### Future directions

Further validation of EASIX thresholds across diverse populations and OA subtypes is essential. Research should also explore whether EASIX-guided therapies (e.g., anti-inflammatory agents or endothelial protectants) improve outcomes and how this biomarker can inform public health strategies for aging societies.

**Table 2:** – Association between EASIX and High Risk of ASCVD and All-Cause Mortality in Patients with Osteoarthritis

Variables	Model 1	Model 2
**High Risk of ASCVD**	**OR (95%CI)**	**OR (95%CI)**
Log (EASIX)	1.97 (1.57-2.48)***	1.94 (1.57-2.41)***
Log (EASIX)		
< -1.29	Ref	Ref
-1.29 - -0.78	1.38 (0.97-1.96)	1.32 (0.93-1.89)
>-0.78	2.44 (1.77-3.35)***	2.31 (1.68-3.18)***
**All-Cause Mortality**	**HR (95%CI)**	**HR (95%CI)**
Log (EASIX)	1.84 (1.34-2.54)***	1.59 (1.14-2.23)**
Log (EASIX)		
< -1.29	Ref	Ref
-1.29 - -0.78	1.38 (0.97-1.96)	1.32 (0.93-1.89)
> -0.78	2.44 (1.77-3.35)**	2.31 (1.68-3.18)*

EASIX: endothelial activation and stress index; ASCVD: atherosclerotic cardiovascular disease; OR: odds ratio; HR: hazard ratio; CI: confidence interval; Ref: reference. Model 1: Crude model. Model 2: Adjusted for education level, physical activity, drinking status, cancer, NLR, and anti-hyperlipidemic agents (for ASCVD) or muscle relaxants and high-risk ASCVD (for mortality). ***p < 0.001, **p < 0.01, *p < 0.05.

## * Supplemental Materials

For additional information Supplemental Table 1, please click here.



For additional information Supplemental Table 2, please click here.



For additional information Supplemental Table 3, please click here.



For additional information Supplemental Table 4, please click here.
